# Is Ghana Prepared for Another Arboviral Outbreak? Evaluating the 2024 Dengue Fever Outbreak in the Context of Past Yellow Fever, Influenza, and COVID-19 Outbreaks

**DOI:** 10.3390/tropicalmed10070196

**Published:** 2025-07-15

**Authors:** Godfred Amoah Appiah, Jerry John Babason, Anthony Yaw Dziworshie, Abigail Abankwa, Joseph Humphrey Kofi Bonney

**Affiliations:** Department of Virology, Noguchi Memorial Institute for Medical Research, College of Health Sciences, University of Ghana, Legon, Accra P.O. Box LG 581, Ghana; gaappiah@noguchi.ug.edu.gh (G.A.A.); jjbabason@noguchi.ug.edu.gh (J.J.B.); aydziworshie@ug.edu.gh (A.Y.D.); aabankwa@noguchi.ug.edu.gh (A.A.)

**Keywords:** arbovirus, outbreak response, outbreak preparedness, Dengue fever virus

## Abstract

Arboviruses are a growing concern in many nations. Several reports of arboviral outbreaks have been recorded globally in the past decade alone. Repeated arboviral outbreaks in developing countries have consistently highlighted vulnerabilities in disease surveillance and response systems, exposing critical gaps in early detection, contact tracing, and resource allocation. The 2024 Dengue fever outbreak in Ghana, which recorded 205 confirmed cases out of 1410 suspected cases, underscored the urgent need to evaluate the country’s preparedness for arboviral outbreaks, given the detection of competent vectors in the country. A retrospective analysis of Ghana’s 2009–2013 pandemic influenza response plan revealed significant deficiencies in emergency preparedness, raising concerns about the country’s ability to manage emerging arboviral threats. This review assessed Ghana’s current arboviral outbreak response and preparedness by examining (a) the effectiveness of vector control measures, (b) the role of early warning systems in mitigating outbreaks, (c) laboratory support and diagnostic capabilities, and (d) community engagement strategies. It highlights the successes made in previous outbreaks and sheds light on several gaps in Ghana’s outbreak response efforts. This review also provides recommendations that can be implemented in many countries across Africa as they brace themselves for any arboviral outbreak.

## 1. Introduction

Arboviral diseases, transmitted primarily by infected arthropods such as mosquitoes, ticks, and sandflies, have emerged as a global health concern [1]. The family of viruses within the arbovirus group—*Flaviviridae*, *Togaviridae*, *Bunyaviridae*, and *Reoviridae*—share a common RNA-based genome, which facilitates rapid adaptation to evolving environmental and host conditions and drives the recent expansion of these viruses into new geographic territories [2].

Dengue virus, a member of the *Flaviviridae* family, is a small icosahedral enveloped virus containing 11 kilobases of positive-sense single-stranded RNA and seven non-structural (NS) proteins: NS1, NS2A, NS2B, NS3, NS4A, NS4B, and NS5 [3]. It is transmitted primarily by the *Aedes aegypti* and *Aedes albopictus* mosquitoes and is endemic in urban, peri-urban, and rural areas across the tropics [4,5]. The alarming global burden of Dengue fever has been linked to several factors, including climate change, urbanization, globalization, and ecological imbalances [6]. The geographic ranges of vectors increase as temperatures rise and precipitation patterns change, enabling these disease carriers to invade previously unaffected areas. Additionally, the uncertainties of climatic changes, such as rainfall patterns in some areas, could have a significant impact on the dynamics of vector populations, thereby creating the conditions for outbreaks [7].

Several incidences of *Aedes*-borne diseases such as Zika virus disease, Chikungunya fever, and Dengue fever have been reported globally in the past decade alone [8]. Notable examples include the interconnected outbreak of Yellow Fever (YF) epidemics in Angola and the Democratic Republic of Congo in 2016, the concurrent Dengue fever and Zika epidemics in South America [9], the Dengue fever outbreak in Malaysian cities, and the Dengue fever outbreak in the French Caribbean [10]. While most countries in the West African sub-region have experienced multiple arboviral outbreaks, Ghana has primarily reported YF cases [11,12,13,14] and has documented Zika virus exposure within its borders [15]. However, the recent declaration of a Dengue fever outbreak by Ghana’s Ministry of Health marks a pivotal shift. Ghana’s tropical climate is conducive to vector proliferation, and identifying potential arbovirus vectors underscores the country’s vulnerability to these emerging threats [13,16].

Ghana’s response to the COVID-19 pandemic, influenza, and YF, among others, demonstrates its public health capacity [12,17]. However, the potential emergence and re-emergence of arboviral outbreaks other than YF, considering neighboring countries’ experiences, necessitate enhanced and well-concerted surveillance, prevention, and response efforts [18]. Early detection of abnormal health events, which could result in epidemics or even pandemics, continues to pose a significant challenge [19]. By examining the successes and shortcomings of past outbreaks, this current review seeks to identify critical gaps in the country’s public health infrastructure in preparation for arboviral outbreaks and provide recommendations for improvement.

## 2. Background Observation

**Figure 1:**
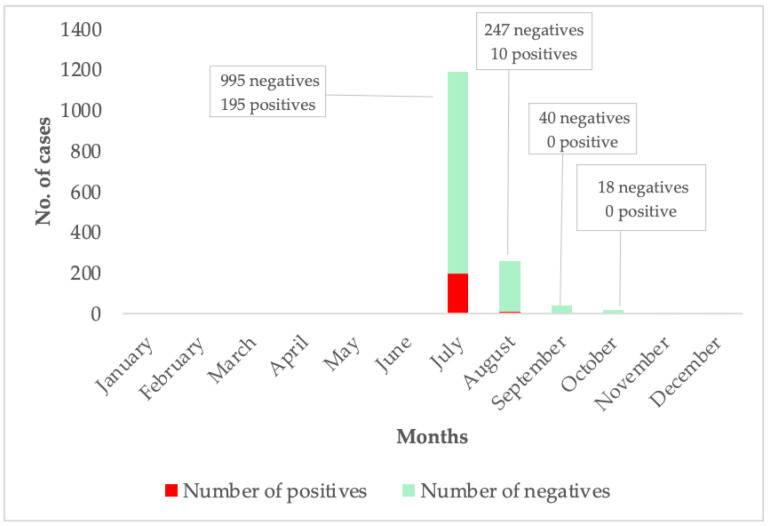
This graph shows the case per month of Dengue fever suspected and confirmed cases. The months where no suspected or confirmed cases are shown above reflect periods during which no samples were submitted for testing, or testing yielded no positive results.

In 2024, reports of unusual cases of malaria-like illness in several districts of the Eastern Region of Ghana that were not responding to standard anti-malarial treatment regimens were detected. These cases were therefore managed as acute febrile illness. Patient samples were sent to the Noguchi Memorial Institute for Medical Research (NMIMR) for further testing. The criteria for suspected Dengue fever cases included acute onset of high fever (≥38 °C) lasting 2–7 days, accompanied by two or more of the following: diarrhea, fatigue, nausea/vomiting, loss of appetite, muscle and joint pains, rash, and haemorrhagic manifestations. The samples were screened for Dengue fever, Chikungunya, and Zika viruses using the CDC Trio plex assay—a real-time quantitative polymerase chain reaction (RT-PCR). Testing confirmed Dengue fever cases. All confirmed cases were classified as non-severe and managed at home. The Ghana Health Service (GHS) declared a Dengue fever outbreak on 14 July 2024. Following the outbreak, 1410 suspected blood samples were tested by NMIMR, of which 205 tested positive for the Dengue fever virus. The confirmed cases were distributed across eight administrative regions of Ghana, with the Eastern Region emerging as the hotspot of the outbreak. The peak of the outbreak was in July (Figure 1). The initial diagnosis of febrile illnesses as malaria is a systemic issue in low- and middle-income countries in the tropics, which are endemic malaria regions, and this impacts the true rate of clinical malaria [19].

## 3. Review of Previous Outbreaks in Ghana

### 3.1. Yellow Fever Outbreaks

Despite a relatively high national YF vaccination rate [20], Ghana has experienced recurrent YF outbreaks, with the most recent one occurring in 2021–2022 [11]. It is reported that YF recurs in Ghana in a 5-year cycle [12]. Several factors have been highlighted as plausible drivers of YF recurrence: environmental drivers [21], vector diversity and capacity, climate change [13], and inaccurate laboratory diagnosis [22]. In 2015, the West Gonja district in the Savannah region was the epicenter of a YF outbreak among unvaccinated nomadic populations, resulting in three deaths among twelve confirmed cases [22]. Sporadic incidences of YF have been reported in the country. According to a World Health Organization (WHO) assessment, Ghana was ranked alongside twenty-seven other African countries as having a high risk of YF outbreak [15]. Increased vaccination efforts against YF were therefore necessary in response. From November 28 to 4 December 2018, Ghana, in partnership with WHO, GAVI, and the United Nations International Children’s Emergency Fund (UNICEF), launched a subnational vaccine campaign that immunized 5.3 million individuals against YF, with a focus on those aged 10 to 60 [16]. However, the recent and severe outbreak from October 2021 to February 2022 still outscores the nation’s outbreak response gap. The outbreak impacted four administrative regions and claimed thirty-five lives out of seventy confirmed cases, resulting in a 50% case fatality rate [12]. Vaccination has proven to be a highly successful method of YF prevention, as more than 80% of people who receive the vaccine develop lasting immunity [20]. However, there is still a portion of the Ghanaian population that is unvaccinated: Fulani pastoral nomads living in remote regions, many of whom are young people actively moving across vast areas of remote territories with their herds of cattle in pursuit of pastures, water, improved living conditions, or just safer surroundings [17,23]. Being outside of the district during the vaccine campaigns, not having access to the vaccination site, and not knowing much about the campaign were the primary reasons for vaccine hesitancy among the nomadic population [20]. Aside from the unvaccinated nomadic population, other probable causes of the recent YF outbreak in the northern region of Ghana can be attributed to spillover from Burkina Faso [15] and proximity to the Mole National Park, where non-human primates and mosquitoes could sustain the sylvatic and savanna cycles of YF [11].

### 3.2. The COVID-19 Pandemic

Ghana recorded two initial cases of COVID-19 on 12 March 2020. Following the surge in case counts, the country went into a partial lockdown for sixteen days, which was subsequently extended for another twenty-two days until 19 April 2020 [24]. The government’s five-pronged strategy to combat the pandemic included prevention of spread, containment, care for the infected, socio-economic impact mitigation, and enhancement of domestic production of personal protective equipment [24]. Implementing measures such as partial lockdowns, border closures, flight suspensions, social gathering bans, and school closures supplemented these goals [25]. The government also expanded laboratory capacity beyond NMIMR and Kumasi Centre for Collaborative Research in Tropical Medicine (KCCR) through increased staffing, 24 h operations, contact tracing, and collaboration with additional facilities like the Veterinary Services Directorate. These efforts expedited testing and reduced sample turnaround times [25,26].

As of April 2020, Ghana had distinguished itself as the only African country to conduct over 60,000 tests for suspected cases of COVID-19 [27]. The country achieved the highest ranking on the continent for tests administered per million people [24]. Ghana effectively managed its vaccine stockpile, with less than 5% of doses expiring before use [28]. The country recorded 94,011 confirmed cases and 785 deaths by May 2021 [29]. Non-governmental organizations supported the COVID-19 public education campaign by creating and distributing safety information through billboards and other communication materials. The Ghana COVID-19 National Trust Fund, which the government created to serve as a conduit for the pooling of non-governmental sector resources to support the government’s COVID-19 response operations, received a substantial contribution from the private sector [25]. Additionally, the COVID-19 outbreak response benefited from technical support provided by retired clinical and public health professionals [29].

However, the fight against COVID-19 encountered considerable obstacles. The inconsistent and insufficient supply of logistics, including testing reagents and consumables, personal protective equipment (PPE), unpaid allowances for frontline healthcare workers, and limited ultra-cold storage facilities, posed significant challenges to the response efforts [28,30]. In addition to straining the already precarious logistic networks, the COVID-19 pandemic created an unforeseen demand for necessary equipment. The manufacturing gap may explain the perceived lack of readiness of the health system in Ghana since healthcare workers lacked the resources needed to effectively contain the pandemic. However, the impending problem was avoided by the local production of PPE, drawing on indigenous ideas to alleviate its shortages in the case of another outbreak, at least temporarily [31].

### 3.3. Pandemic Influenza Virus Outbreaks

In 2007, Ghana launched a countrywide monitoring system backed by the United States Naval Medical Research Unit No. 3 (NAMRU-3), now the NAMRU-EURAFCENT, and the GHS for influenza-like illnesses (ILIs) in response to the highly pathogenic avian influenza A *H5N1* outbreak among poultry [32]. Between 2009 and 2010, numerous cases of acute febrile respiratory illnesses were reported nationwide, with the highest proportion of positive cases being children of school-going age [33,34]. Again, an influenza A(H1N1) pdm09 outbreak (Case Fatality Rate = 4.2%) was also recorded in a Ghanaian secondary school in 2019 [35]. In another school setting, 9 out of 17 (52.9%) nasopharyngeal swabs collected from suspected cases tested positive for influenza A(H1N1)pdm09 [36].

Ghana’s influenza surveillance system has been valuable over the past two decades by identifying and categorizing virus strains and providing essential data to guide public health decisions and interventions [37]. However, the re-emergence of influenza outbreaks raises questions about the rapid evolution of the influenza virus and the need for increased surveillance. Ghana’s approach and preparedness towards pandemic influenza were guided by frameworks recommended by the WHO and the Food and Agriculture Organization (FAO). The strategy focused on five critical areas: planning and coordination; surveillance and situation monitoring; prevention, containment, and management; communications; and social mitigation [38,39]. The overarching goal was to build on the response systems previously established for severe acute respiratory syndrome (SARS) and avian influenza, ensuring the country was equipped to detect and manage any future influenza pandemics effectively [40]. An evaluation by Nuvey and colleagues in 2019 revealed that the ILI sentinel surveillance system in Greater Accra, while vital for the early detection of new influenza viruses and representative of the local population, did not meet its objectives. In their review, they observed that the sentinel locations often fell short of their yearly case detection quotas, lacked thresholds for alerting the health system, and did not conduct antiviral resistance testing for isolates [37].

## 4. Current Preparedness Assessment

Repeated outbreaks of infectious diseases in developing nations have consistently exposed and highlighted the vulnerabilities of disease surveillance and response systems in developing countries, revealing critical gaps in early detection, contact tracing, and resource allocation [41].

A retrospective analysis of Ghana’s 2009–2013 influenza response plan was conducted to assess the preparedness of sub-Saharan African nations for health emergencies. The findings revealed that the country’s emergency preparedness was in disarray [40]. To assess the current preparedness of Ghana concerning arboviral threats, we seek to investigate (a) the effectiveness of vector control measures as part of surveillance and monitoring efforts in Ghana, (b) the role of early warning systems in predicting and mitigating arbovirus outbreaks, (c) laboratory support and preparedness, and (d) community engagement.

### 4.1. (a) Vector Control Effectiveness

The presence of *Aedes* mosquitoes, particularly *Aedes aegypti*, has been well-documented as competent vectors necessary for the transmission of arboviruses, including the Dengue fever virus, in Ghana [42,43,44]. An entomological surveillance of the larval indices in Ghana estimated that the population density of *Aedes aegypti* in Cape Coast was sufficient to cause an arboviral outbreak [45]. The large population of these vectors in the country is linked to the presence of water-holding containers near human habitats, which act as breeding sites for these mosquitoes, as well as urbanization [46,47]. Over time, *Aedes aegypti* has adapted to thrive close to humans, often preferring to feed almost exclusively on them, even when other potential hosts are available [48]. With various morphological and behavioral adaptations, including feeding on various blood sources or producing eggs that can tolerate desiccation, skip oviposition, and exhibit dormancy, they can also withstand extraordinary climatic stressors. Furthermore, *Aedes* vectors have evolved in recent years to lay their eggs in uncommon breeding environments, like salty and organically contaminated water. As a result, in the ever-changing world of today, *Aedes* vectors have evolved into clever survivors [49]. Several control measures can be adapted to control the vector population in Ghana. Enhancing the quantity and consistency of the water supply, as well as the sanitation conditions, could therefore be a significant control intervention. This aligns with the WHO’s guidelines for controlling *Aedes aegypti* [50]. Building on the success of *Anopheles* mosquito control strategies in the country, which contributed to a 40% reduction in falciparum malaria cases over 15 years in sub-Saharan Africa [51], a similar concerted effort is needed to address the growing challenge of *Aedes* mosquito populations in Ghana.

Another concern is vector resistance, which is noted to thwart efforts to reduce the vector population. *Aedes* mosquito populations have been found to exhibit a variety of sodium channel mutations and associated pesticide resistance in studies conducted globally and locally [52,53]. For example, Suzuki et al. found resistance against lambda-cyhalothrin and DDT in insecticides [43]. Also, a study by Kudom [45] found varying levels of resistance against pyrethroid insecticides. Integrated vector management (IVM) approaches, including environmental management, biological control, and the judicious use of insecticides, should be prioritized to target *Aedes* populations effectively [43]. The IVM approach places a strong focus on using the right mosquito control tools, assessing and evaluating the local situation, and making decisions at the decentralized level [54]. These approaches can be implemented in hotspot areas in Ghana to aggressively reduce vector populations.

Community engagement is a vital component of these strategies. Public education campaigns about removing water-holding containers and improving sanitation can significantly reduce breeding sites [51]. Strategies aimed at reducing the Aedes mosquito population can be implemented through biological, chemical, or physical manipulation techniques that disrupt the life cycle stages of *Aedes aegypti*, particularly by targeting their larvae in water bodies and controlling adult populations [55,56]. Biological control methods involve the use of natural predators or microbial agents to reduce mosquito larval populations. For instance, larvivorous fish can be introduced into water bodies, where they feed on mosquito larvae and effectively reduce their numbers [43]. In addition, bacterial larvicides such as *Bacillus thuringiensis israelensis* (Bti) and *Bacillus sphaericus* can be applied. These bacteria produce toxins that are specifically lethal to mosquito larvae but are harmless to most non-target organisms, including humans. When used at recommended rates, Bti typically achieves 90–100% mortality of target mosquito larvae, with minimal or no direct impact on non-target aquatic and terrestrial organisms [57]. Chemical control methods include the use of larvicides such as pyriproxyfen and methoprene, which interfere with the normal growth and development of mosquito larvae. Other agents, such as oils and surface films, act by forming a barrier on the water surface, suffocating the larvae [58]. Physical manipulation of the larval environment is another straightforward and effective strategy. This includes the elimination of potential breeding sites, such as stagnant water containers, which can be achieved through community involvement and public awareness campaigns [43]. These methods have shown potential in pilot studies conducted in several countries and could be adapted for use in Ghana with appropriate regulatory frameworks and community acceptance [59].

### 4.2. (b) Early Warning Systems in Predicting and Mitigating Arbovirus Outbreaks

About 2.5 billion people reside in regions where *Aedes*-borne illnesses are a concern, and an estimated 390 million infections occur yearly across 100 nations. Risk projections suggest that over the 21st century, these epidemics will worsen and spread to new regions [60]. While the response to the 2024 Dengue fever outbreak may have been swift, it was largely reactive, driven by the surge in active cases rather than the predictive capabilities of an established early warning system (EWS) [61].

The EWS can be divided into two types: those based on traditional surveillance data (using temporal, spatial, or dynamic models) and those using multi-source data (employing artificial intelligence techniques). Examples of traditional models include compartment, Hawkes, regression, and Markov chain models, while multi-source models include random forest, artificial neural networks, conventional neural networks, and recurrent neural networks [62]. The primary goal of these EWS tools is to prevent or mitigate disease outbreaks. An effective arbovirus surveillance system should reliably forecast the timing and location of potential outbreaks [62]. For outbreak EWS, nations must have standard operating procedures (SOPs) to reliably detect an elevated outbreak risk in time and space through alarm signals, prompting an early reaction [60]. In several places, studies on EWS have been carried out for arboviruses [52,53,61,63,64,65]. A 4-year citywide study in Brazil used an integrated surveillance system, which included entomologic, epidemiologic, and entomo-virologic data, and showed that adult mosquito trapping provided a more reliable alert signal of Dengue fever outbreaks than widespread traditional indices based on larval surveys. The study asserted that such early detection could offer local health officers approximately a month to promote and intensify vector control in areas with higher risk [63]. Additionally, in a systematic review conducted on integrated disease surveillance and response in Sub-Saharan Africa, most countries in the sub-Saharan region rely keenly on traditional indicator-based disease surveillance by utilizing existing data from healthcare facilities [66]. In Ghana, the surveillance system operates within the Integrated Disease Surveillance (IDSR) framework, adapted from guidelines established by the WHO’s Regional Office for Africa [67]. Entomological studies [9,48,68,69] have predominantly focused on the use of mosquito traps and human landing catches to survey larval and adult mosquitoes. However, early warning systems incorporating climate data, remote sensing, and vector surveillance have proven essential in predicting and mitigating arbovirus outbreaks. Rainfall anomaly maps, for instance, have been successfully used in malaria early warning systems, demonstrating the potential of environmental monitoring in forecasting arbovirus risks [70].

To address this gap, Ghana and other *Aedes*-endemic countries could benefit from integrating advanced technologies and data-driven approaches into their surveillance systems. For instance, combining entomological indices with modern tools such as geographic information systems (GISs) [53] and machine learning models [64] could enhance the ability to predict high-risk areas and map vector habitats, monitor climate variables, and analyze outbreak patterns [71]. Additionally, strengthening and integrating community-based surveillance and training of community health workers and non-governmental organization staff on EWS protocols can play a pivotal role in enhancing outbreak preparedness [72]. Moreover, regular testing of mosquito populations for viral presence and monitoring of human seroprevalence [73] can act as critical alarm signals for impending outbreaks.

### 4.3. (c) Laboratory Support and Preparedness

Access to rapid and reliable laboratory diagnostics is a critical factor in effectively controlling an infectious disease outbreak; post-outbreak analyses indicate that diagnosing 60% of patients within 1 day rather than 5 days could have decreased the attack rate from 80% to almost 0% in the West African Ebola epidemic of 2013–2016 [74]. The Ebola outbreak scare revealed a significant gap in our laboratory preparedness. A study conducted in Ghana revealed that Ghanaian health personnel were not adequately equipped to handle the viral outbreak. The researchers also expressed concern about the potential lack of proactive response from healthcare workers in other outbreaks, which calls for a coordinated strategy for management and preparation [75].

To respond to outbreaks effectively and appropriately, it is not enough to merely supply the necessary resources, such as drugs, PPE, and other logistical support. It is reported that healthcare workers who have experienced outbreaks are confident in tackling the next outbreak [76]. Existing laboratories at NMIMR, KCCR, and the National Public Health Reference Laboratory in Ghana need to be continually supported by the government while new facilities are constructed to ease the workload. The country needs more robust laboratory systems beyond the reference laboratories to bolster active and passive laboratory surveillance programs [76]. This includes the expansion of testing tools, including NS1 antigen detection, Enzyme-linked immunosorbent assay (ELISA), and the creation of adaptable diagnostic platforms that can quickly implement testing for various infections. This is one strategy for creating sustainable facilities at the national level. Multi-pathogen panels are especially useful for the early identification and tracking of pathogens that cause outbreaks. This enables prompt action without placing an excessive burden on human resources by diverting focus from other duties, requiring more training, or necessitating the establishment of new supply chains [74]. Sentinel surveillance programs that integrate laboratory diagnostics provide critical data to inform public health interventions and policy decisions [77]. Investing in laboratory personnel through training programs is essential for ensuring preparedness. Equipping laboratory staff with skills in molecular diagnostics, bioinformatics, and quality control can enhance diagnostic accuracy and reliability [78]. International partnerships and collaborations with organizations like the Centers for Disease Control and Prevention (CDC), World Health Organization (WHO), and other standard laboratories need to be strengthened to provide Ghanaian laboratories with access to cutting-edge techniques and resources [77].

### 4.4. (d) Community Engagement

A more robust health workforce is required to withstand a sudden surge in deadly infectious diseases in sub-Saharan Africa (SSA) [78]. Reports from USAID observed that the region lacks 2.4 million healthcare workers, and the WHO estimates an increase to 6.7 million by 2030. Considering these shortages, it is important to mobilize community health workers who have demonstrated potential in SSA over the past few decades to augment medical and health service workforces [78]. It is necessary to consider how people interact and coexist through their historical pathways and structures when deciding how best to respond to and adapt to disease outbreaks. For instance, societal norms and beliefs, as well as variations in political, cultural, and social structures, institutions, and processes within the community, influence health behaviors and outcomes during outbreaks [79]. A lack of confidence in health officials and the propagation of rumors among communities can hinder efficient responses to outbreaks and pandemics, according to lessons learned from previous pandemics, such as the 2014 Ebola virus disease outbreaks and the COVID-19 pandemic [80]. Effective community engagement strategies can encourage acceptance and adherence to non-pharmaceutical interventions that can help stop the spread of infectious diseases, like contact tracing, physical separation, and lockdowns [81]. In the absence of vaccines or treatments, these steps are crucial for reducing the spread of infectious diseases because they help break individual chains of transmission and stop outbreaks. Additionally, community engagement is crucial as it gives people access to the resources and information they need to safeguard their neighborhoods and themselves [80]. Also, outreach services by health workers away from their usual workplace and virtual services such as telemedicine or mobile-phone-based approach offer opportunities for remote surveillance, data collection, and disease management among hard-to-reach demographics like nomadic populations [82].

Internationally significant public health situations demonstrate the necessity of culturally relevant community engagement tactics [83]. One such approach involves mobilizing community health workers (CHWs), who have proven invaluable in augmenting SSA medical workforces. CHWs are often members of the communities they serve, which enables them to navigate cultural norms, foster trust, and deliver healthcare services effectively [84].

To further enhance the impact of community engagement in health emergencies, the integration of social science expertise is crucial. Social scientists can provide specialized knowledge in analyzing the social, cultural, historical, political, and economic contexts of affected communities and actors involved in emergency response efforts [85]. By fostering communication and building bridges in challenging situations, social scientists can help redesign interventions to promote greater community ownership and sustainability. For example, in the Ebola outbreak in West Africa, social scientists were instrumental in addressing resistance to public health measures by tailoring interventions to local, cultural, and social dynamics [85].

To successfully integrate these strategies into the current health infrastructure, community engagement must prioritize listening to stakeholders, finding opportunities for discussions, and creating the connections and circumstances necessary for this to happen. It is crucial to ensure that these strategies have an immediate and direct influence on public health initiatives in the population [86]. This alignment between research and community needs can facilitate the development of culturally and epidemiologically appropriate solutions, thereby improving health outcomes and resilience in vulnerable populations [74].

## 5. Future Perspectives

The assessment of Ghana’s preparedness for arboviral outbreaks reveals several critical areas that warrant future attention and development. Ghana needs to develop a comprehensive, real-time surveillance platform that integrates clinical, laboratory, and entomological data. Advanced predictive modeling using artificial intelligence and machine learning can be implemented to understand transmission dynamics and forecast potential outbreaks. To effectively control the spread of mosquito-borne pathogens, a strong knowledge of the vector population and characteristics needs to be available. For this purpose, long-term entomology laboratory studies are needed. The government should also prioritize the expansion of molecular diagnostic capabilities to regional laboratories to augment outbreak response efforts. Ghana should collaborate with neighboring countries and foreign bodies to strengthen cross-border surveillance networks.

## 6. Conclusions

The emergence and re-emergence of arboviral diseases in Ghana, exemplified by the recent Dengue fever outbreak, underscore the urgent need to strengthen the nation’s public health infrastructure. While previous responses to outbreaks like Yellow Fever, COVID-19, and influenza have demonstrated resilience, critical gaps remain in vector control, early warning systems, and laboratory preparedness. Addressing these gaps requires an integrated approach that combines robust surveillance systems, innovative vector management strategies, and enhanced laboratory support. Community engagement and international collaborations will also be pivotal in building a comprehensive framework to mitigate future outbreaks. We also recommend an integrated approach that uses data from humans, animals, and the environment (One Health Approach) to predict, identify, and quell potential outbreaks. By leveraging lessons from past epidemics and embracing modern technological advancements, Ghana can bolster its preparedness and response capabilities to safeguard public health against the growing threat of arboviral diseases.

## Abbreviations

The following abbreviations are used in this manuscript:

CDC	Centers for Disease Control and Prevention
CHWs	Community Health Workers
COVID-19	Coronavirus Disease 2019
ELISA	Enzyme-Linked Immunosorbent Assay
EWS	Early Warning Systems
FAO	Food and Agriculture Organization
GHS	Ghana Health Service
GIS	Geographic Information Systems
IDSR	Integrated Disease Surveillance
ILI	Influenza-Like Illnesses
IVM	Integrated Vector Management
KCCR	Kumasi Centre for Collaborative Research in Tropical Medicine
KNUST	Kwame Nkrumah University of Science and Technology
NAMRU-3	U.S. Naval Medical Research Unit No. 3
NMIMR	Noguchi Memorial Institute for Medical Research
NS	Non-Structural
PPE	Personal Protective Equipment
RNA	Ribonucleic Acid
RT-PCR	Real-Time Quantitative Polymerase Chain Reaction
SARS	Severe Acute Respiratory Syndrome
SOPs	Standard Operating Procedures
SSA	Sub-Saharan Africa
UNICEF	United Nations Children’s Fund
USAID	United States Agency for International Development
WHO	World Health Organization
YF	Yellow Fever
